# Restoring glucose metabolic homeostasis to attenuate ovarian aging: mechanisms and clinical prospects

**DOI:** 10.1186/s13048-026-02244-1

**Published:** 2026-08-29

**Authors:** Genping Zeng, Mengyuan Chu, Jiaqi Yang, Yunfei Han, Qianyi Zhou, Han Zhang, Ying Yan

**Affiliations:** 1 First Teaching Hospital of Tianjin University of Traditional Chinese Medicine, Tianjin, 300381 China; 2 National Clinical Research Center for Chinese Medicine, Tianjin, 300381 China

**Keywords:** Ovarian Aging, Glucose Metabolism, Metabolic Homeostasis, Post-translational Modifications, Metabolic Reprogramming, Therapeutic Target

## Abstract

Ovarian aging has emerged as a major challenge to female reproductive health worldwide, primarily characterized by a decline in oocyte quality and/or quantity, diminished granulosa cell function, and impairment of the surrounding microenvironment. Within this microenvironment, glucose metabolism participates in the synergistic regulation of ovarian homeostasis through specific metabolic networks and metabolite-driven signaling. Metabolic disruption can impair intercellular communication mechanisms, induce oxidative stress and mitochondrial dysfunction, and ultimately drive ovarian aging. Ovarian granulosa cells surround the oocyte and facilitate bidirectional communication via gap junctions or molecular signaling. These cells continuously supply energy substrates to support oocyte growth and maturation, while the oocyte, in turn, promotes granulosa cell proliferation and differentiation. Impairment of glycolysis in granulosa cells leads to energy deficits and triggers apoptosis. Concurrently, metabolic reprogramming in the oocyte links glucose metabolic homeostasis with mechanisms governing developmental competence. Glucose-derived metabolites drive various post-translational modifications (PTMs), including glycosylation, acetylation, phosphorylation, and succinylation, that contribute to ovarian function and intersect with pathological processes such as oxidative stress, chronic inflammation, and autophagy. Therefore, therapeutic strategies targeting glucose metabolism, such as modulating metabolic pathways, metabolite signaling, and associated PTMs, offer promising avenues for treating ovarian aging. However, the glucose metabolic network within the ovarian microenvironment is highly complex, and targeting a single node is often insufficient to restore metabolic homeostasis. A comprehensive approach that integrates multiple strategies to simultaneously intervene at different regulatory levels is needed. This review summarizes recent advances in understanding the ovarian glucose metabolic network, metabolite-driven post-translational modifications, and the interplay among various pathological mechanisms. We further explore potential regulators that could restore glucose metabolic balance and propose that future efforts should focus on developing precision strategies, guided by artificial intelligence and multi-omics data to modulate the metabolic reprogramming of both the microenvironment and functional cells, ultimately translating these insights into clinical interventions for ovarian aging.

**Graphical Abstract:**

**Figure:**
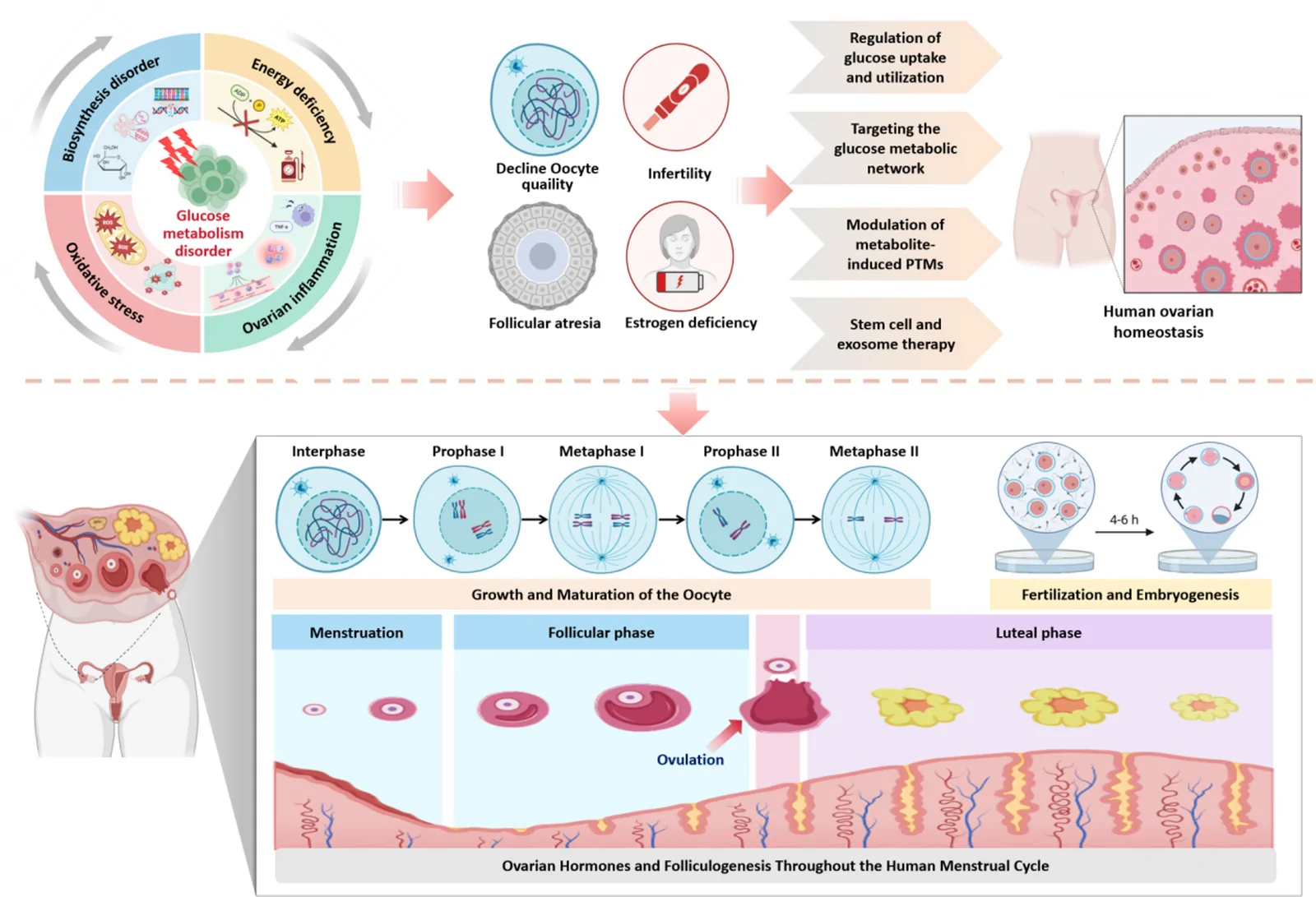

## Introduction

**Fig. 1:**
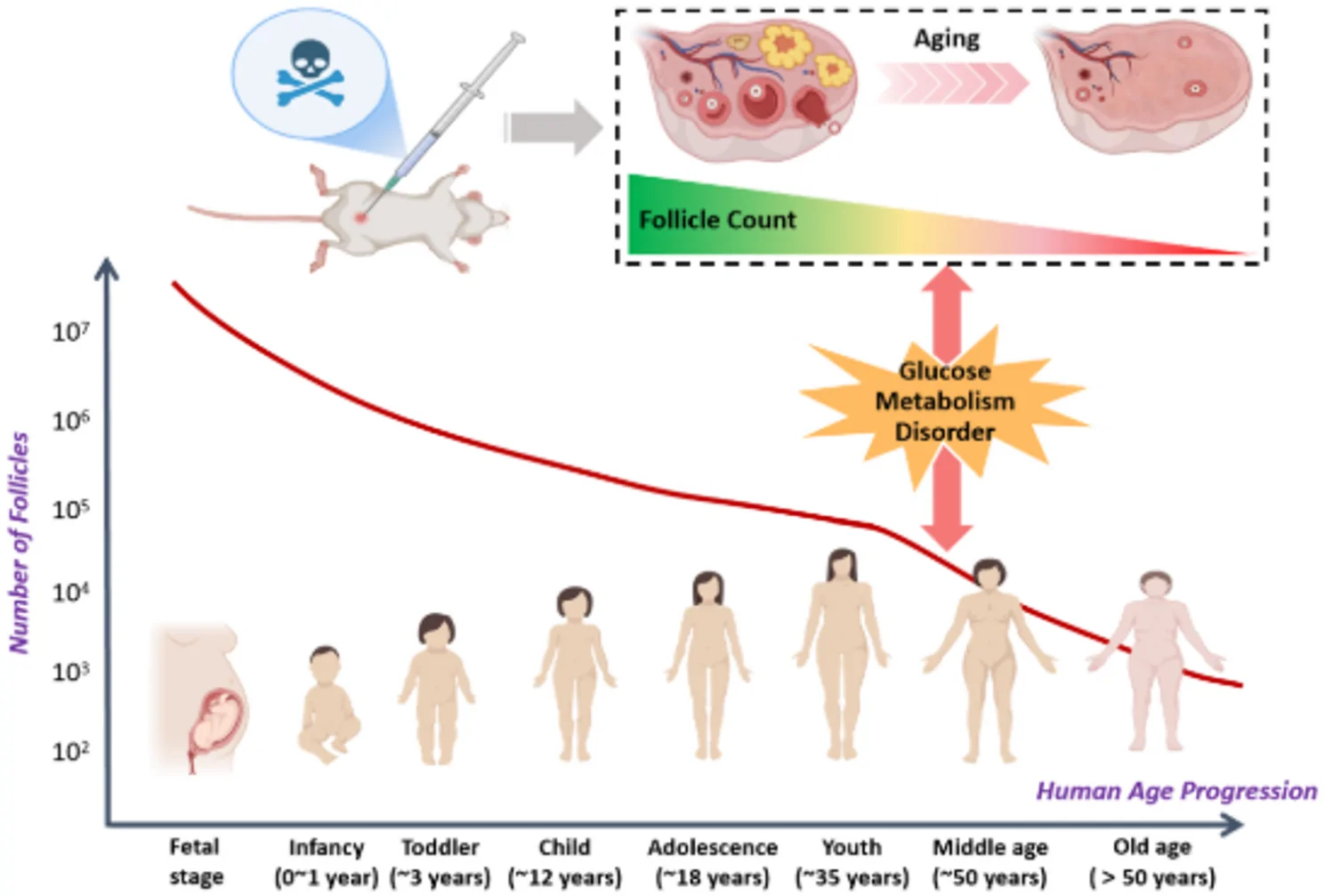
Schematic diagram of physiological and pathological ovarian aging. The number of follicles gradually decreases with age or due to pathological factors, among which glucose metabolism disorder is a key driver. The figure was created using BioRender

**Fig. 2:**
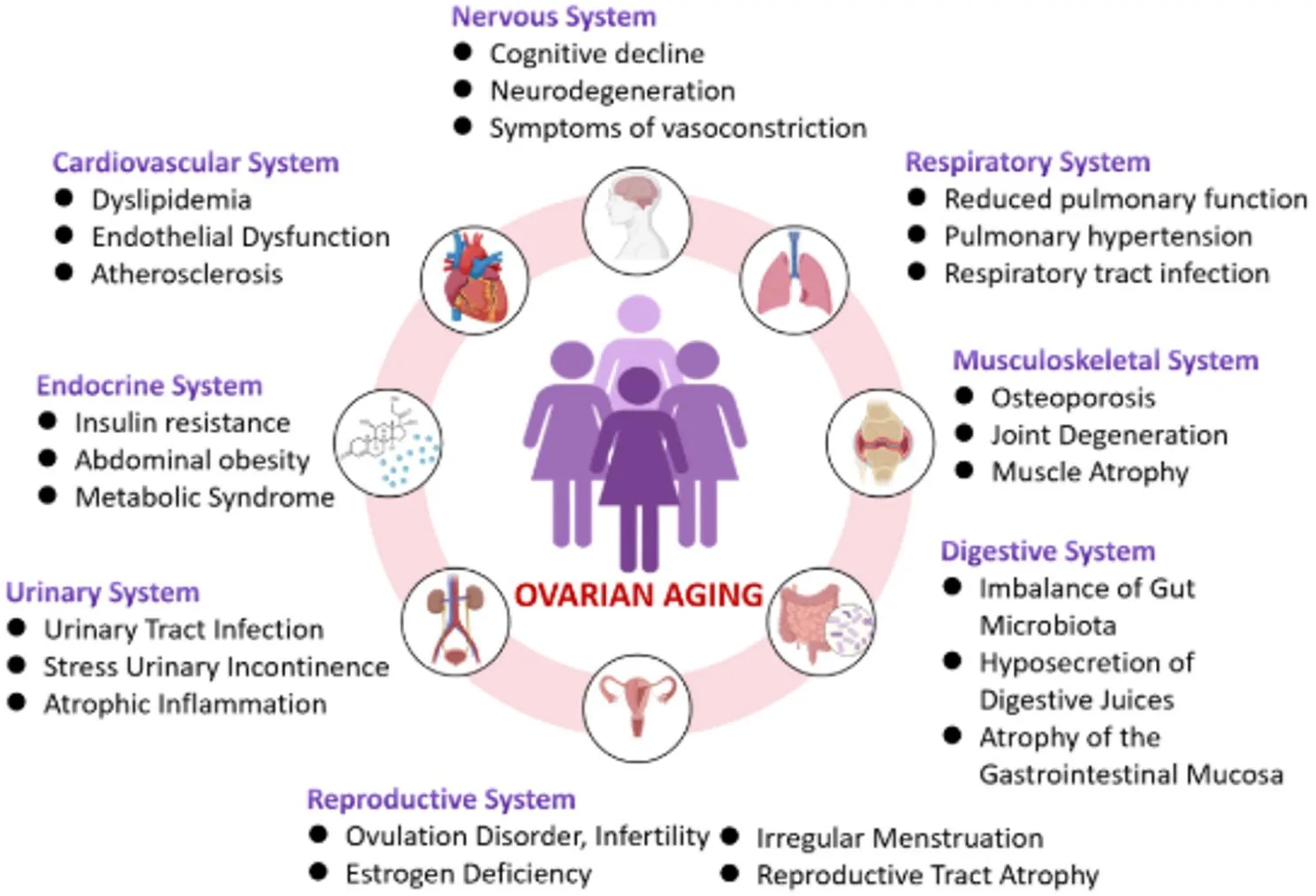
The association between ovarian aging and systemic pathological changes across various organ systems. The figure was created using BioRender

Ovarian aging is the core sign of the decline in the function of the female reproductive system. It not only affects the entire life course of the body but also intensifies the pressure of population aging, evolving into a global health challenge [1]. Ovarian aging refers to the process in which ovarian function gradually declines and eventually exhausts, which can be divided into physiological and pathological forms. Physiological aging refers to the natural decline of ovarian function with age until menopause. Pathological aging refers to the premature decline of ovarian function that is not in line with age, including premature ovarian insufficiency (POI), premature ovarian failure, diminished ovarian reserve (DOR), and poor ovarian response [2]. The pathological manifestations include a decrease in the number and/or quality of oocytes, granulosa cells (GCs) impairment, and disorder of the ovarian microenvironment, accompanied by a decrease in estrogen levels [3](Fig. 1). The clinical manifestations include low pregnancy rates, high miscarriage rates, and low live birth rates [4]. Ovarian aging often occurs earlier than other organs in the human body, not only seriously affecting female fertility but also causing pathological changes in multiple systems through estrogen signaling (including cardiovascular diseases, vasomotor symptoms, osteoporosis, and Alzheimer’s disease, etc.) (Fig. 2) [5]. Ovarian aging has nine key markers: mitochondrial dysfunction, oxidative stress, genomic instability, telomere attrition, epigenetic alterations, impaired autophagy, cellular senescence, dysregulated nutrient sensing, and chronic inflammation, forming an interwoven network mechanism that jointly drives the decline of ovarian function. In this complex network, the precise metabolic collaboration between oocytes and surrounding GCs, theca cells, and other somatic cells is particularly crucial [6]. The pathological process of ovarian aging is essentially the damage to the metabolic collaboration network at the levels of energy metabolism, redox balance, and molecular signal communication, especially the disruption of glucose metabolism homeostasis [7, 8]. As the core hub connecting cellular energy supply, biosynthetic demands, epigenetic modifications, and stress responses, it plays a key role in maintaining ovarian structure and function. The utilization of glucose by cells participates in the regulation of physiological homeostasis and pathological mechanisms, and the development of oocytes is not only highly dependent on the surrounding microenvironment but also requires substantial energy expenditure [9]. They secrete factors to support the proliferation of surrounding GCs, and the bidirectional communication between them is the basis of follicular maturation [10]. This bidirectional communication originates from the assembly stage of primordial follicles and runs through follicular development until ovulation [11]. Glucose is not only a typical energy source, but its metabolites are also multifunctional signaling molecules. For example, pyruvate regulates the rate of oxidative phosphorylation (OXPHOS), participates in the mechanisms of GCs apoptosis and oocyte maturation [8]; while lactate, as the final product of glycolysis, regulates oxidative stress and histone lactylation modification, playing an important regulatory role in inflammation, tissue repair, epigenetics, and other fields [1, 12, 13]. In serum of patients with POI, lactate levels were significantly elevated and negatively correlated with ovarian reserve function. Excessive lactate accumulation in GCs of POI mice induced premature follicle development and subsequent depletion. The underlying mechanism may be related to enhance glycolysis and abnormal lactylation [14]. While in DOR, the activity of key glycolytic enzymes (such as pyruvate kinase PKM2 and hexokinase HK2) decreases, promoting reactive oxygen species (ROS) production, which will affect spindle assembly migration, chromosome separation, and embryo development potential in oocytes [15, 16], and the specific molecular mechanisms are often regulated by PTMs mediated by glucose metabolites. Moreover, although the lactate level in granulosa cells of DOR patients shows a decreasing trend, which seems to contradict the previous conclusion, the pathological mechanisms of DOR and POI are fundamentally different. The underlying mechanism in DOR lies in the reduced capacity of cells to take up glucose, making it difficult to generate lactate, whereas in POI, lactate elevation is driven by abnormal lactylation and enhanced glycolysis [17]. Therefore, the disorder of the ovarian microenvironment is an important driving factor for ovarian aging, including the disorder of intercellular metabolic cross-talk, mitochondrial dysfunction, and abnormal nutrient sensing pathways [8, 18, 19].

Nevertheless, the collaborative network of glucose metabolism currently lacks systematic understanding in the process of ovarian aging. A thorough analysis of the ovarian microenvironment, particularly the dynamic changes in the oocyte-somatic glucose metabolism collaboration network during aging, is of fundamental significance for comprehensively understanding the mechanisms of ovarian aging and developing targeted intervention strategies.

## Methods for literature search

We performed a comprehensive search across PubMed, Web of Science, Embase, CNKI, and Wanfang Database from database inception to February 10, 2026. The following keyword combinations were used: (“ovarian aging” OR “ovarian senescence” OR “ovarian dysfunction” OR “premature ovarian insufficiency” OR “diminished ovarian reserve” OR “premature ovarian failure”) AND (“glucose metabolism” OR “glycolysis” OR “oxidative phosphorylation” OR “pentose phosphate pathway” OR “metabolic reprogramming” OR “mitochondrial dysfunction”) AND (“post-translational modifications” OR “acetylation” OR “lactylation” OR “succinylation” OR “glycosylation” OR “methylation” OR “phosphorylation”). Equivalent Chinese terms were applied for the Chinese databases. Original research, systematic reviews, meta-analyses and case reports were included, while conference abstracts, commentaries, and non-English/Chinese articles were excluded. Priority was given to human studies, followed by mechanistic animal studies; correlative findings were interpreted with caution.

## Dysregulation of glucose metabolic homeostasis as a central driver in ovarian aging

### Compartmentalized metabolism: the spatiotemporal crosstalk between gcs and oocytes

Glucose metabolism in the ovary is not a homogeneous process, but rather a highly compartmentalized and cooperative system between GCs and the oocyte [20, 21]. This structural and functional division of labor is fundamental to maintaining ovarian physiology. GCs, the principal somatic cell population surrounding the oocyte to form the follicle, exhibit a high glycolytic flux coupled with active mitochondrial OXPHOS [21]. This energetic profile is supported by the high membrane expression of glucose transporters (e.g., GLUT1, GLUT4), facilitating efficient glucose uptake [22, 23]. The subsequent entry of pyruvate into the mitochondrial tricarboxylic acid (TCA) cycle drives efficient adenosine triphosphate (ATP) synthesis, fueling GCs proliferation, differentiation, and steroidogenesis [24]. Utilizing acetyl-coenzyme A and NADPH generated from glucose metabolism, GCs efficiently convert cholesterol to estradiol under the regulation of follicle-stimulating hormone. Concurrently, they supply key glycolytic and pentose phosphate pathway (PPP) metabolites to the follicular fluid or directly to the oocyte via gap junctions and paracrine signaling [25, 26]. In contrast, the oocyte, despite harboring the highest mitochondrial copy number among human cells, maintains a state of relative metabolic quiescence with a unique energetic strategy. It primarily depends on glycolytic intermediates (e.g., pyruvate, lactate) supplied by the surrounding GCs to meet the bioenergetic demands of meiosis and cytoplasmic maturation [8, 27]. The oocyte itself exhibits limited intrinsic glucose uptake and low OXPHOS activity. This metabolic restraint serves to minimize the generation of reactive ROS from both glycolysis and mitochondrial respiration. Furthermore, the oocyte can engage the PPP to generate ribose-5-phosphate for nucleotide synthesis and NADPH to mitigate ROS-induced damage [28]. Therefore, this precise spatiotemporal metabolic crosstalk is a critical determinant of oocyte developmental competence [29].

However, this metabolic dependency is not a passive process. The oocyte actively orchestrates its own metabolic support system through sophisticated “bidirectional communication.” It maintains close physical contact with GCs via specialized transzonal projections, while its microvilli facilitate the release of oocyte-secreted factors that modulate GCs activity [30]. This dual structural framework forms the basis of their functional interaction. Crucially, the oocyte regulates GCs glycolytic flux by inducing the expression of key glycolytic genes. Additionally, it acts as a “metabolic conductor” through its secretion of specific oocyte-secreted factors, primarily growth differentiation factor 9 (GDF-9) and bone morphogenetic protein 15 (BMP-15) [9, 31]. These factors are indispensable for coordinated follicular development. For instance, treatment of immature rat ovaries with recombinant GDF-9 can induce primordial follicle growth and promote subsequent follicular development [32]. GDF-9 supplementation positively influences folliculogenesis regardless of follicle-stimulating hormone presence [33, 34]. In mice, GDF-9 and BMP-15, expressed exclusively in the oocyte, form a potent heterodimer that activates the expression of genes associated with cell proliferation (e.g., Ccnd2, a key driver of GCs mitosis) [35]. They thereby precisely regulate cumulus cell expansion, differentiation, steroidogenesis, and metabolic activity. Furthermore, BMP-15 synergizes with another oocyte-secreted factor, fibroblast growth factor 8, to upregulate glycolytic genes in GCs and enhance gap junctional communication, ensuring efficient nutrient production and transfer [36]. This reciprocal relationship is fundamental for synchronous development of both cell types. Disruption of this communication network impairs folliculogenesis and contributes to structural and functional ovarian decline. Therefore, the compartmentalized yet interactive metabolic network is a core pillar of ovarian function, and its dysregulation constitutes a key pathological mechanism underlying ovarian aging.

### Pathological alterations in the ovarian microenvironment

The ovarian microenvironment is essential for follicular growth, development, and maturation. Its homeostasis relies on the precise coordination of the extracellular matrix, vascular network, immune regulation, and biochemical signaling [37, 38]. The extracellular matrix provides structural support for follicles, and its mechanical properties influence primordial follicle activation primarily through key pathways such as Hippo and PI3K/Akt [39]. Concurrently, a well-developed vascular network delivers oxygen and nutrients to follicles while facilitating the removal of metabolic waste products. The immune microenvironment, comprising immune cells like macrophages, regulatory T cells, and T helper 17 cells, performs immune surveillance and regulation to maintain inflammatory and immunological balance [40]. Furthermore, reproductive hormones and critical growth factors, including vascular endothelial growth factor and transforming growth factor-beta, participate in the precise, stage-specific regulation of folliculogenesis.

Nevertheless, with advancing age, the ovarian microenvironment undergoes a series of pathological alterations, among which dysregulation of glucose metabolism serves as a pivotal driver [41]. Age-related impairment in cellular glucose uptake and utilization leads to the accumulation of advanced glycation end products (AGEs) [42]. These AGEs alter the recruitment and activation of immune cells, elevating levels of pro-inflammatory cytokines such as tumor necrosis factor-alpha and interleukin-6 in the ovarian stroma [43]. This shifts the immune microenvironment, primarily orchestrated by macrophages, towards a pro-inflammatory state, characterized by a reduction in regulatory T cells and an increase in T helper 17 cell populations, thereby accelerating ovarian functional decline [44, 45]. Furthermore, glucose metabolic disturbances promote ovarian stromal fibrosis by activating fibroblasts and enhancing extracellular matrix synthesis through AGE-mediated signaling and epigenetic modifications, a pathological feature validated in aged mouse ovarian tissues [46]. From a vascular perspective, the ovary is a site of physiological cyclic angiogenesis. The dynamic balance between the initiation and regression of blood vessels is critical for hormone delivery and nutrient supply to follicles, particularly in mature follicles, which are densely vascularized. Pathological dysregulation of this process underlies various female reproductive disorders. Dysfunctional glucose metabolism impairs endothelial cell function, compromising angiogenic capacity and blood perfusion, leading to reduced vascular density and a state of follicular nutrient deprivation. Studies indicate that augmenting the expression of pro-angiogenic factors like vascular endothelial growth factor can improve follicle numbers and oocyte quality [47]. Notably, the recently identified protein CCDC134, involved in immune regulation and apoptosis, has been shown to upregulate vascular endothelial growth factor to promote angiogenesis while also reducing GCs apoptosis by modulating the expression of Bcl-2, Bax, and Caspase-3 [48]. In summary, disruption of glucose metabolic homeostasis drives ovarian aging by inducing a triad of microenvironmental insults: angiogenic failure, chronic inflammation, and fibrotic damage, collectively promoting follicular atresia. The key metabolites involved in ovarian glucose metabolism and their roles are summarized (Tables 1 and 2), highlighting potential intervention points for strategies aimed at delaying ovarian aging. However, given the compartmentalization of metabolic pathways in granulosa cells and oocytes, some studies have not yet clarified the changes in these metabolites and catalytic enzymes specifically within granulosa cells and oocytes. Thus, the conclusions have limitations and require further experimental verification.

**Table 1:** Alterations in Glucose Metabolites and Associated Proteins in Ovarian Aging

Metabolite	Metabolic Pathway Involved	Contents	Sample type	Disease Type	Reference
Glucose	Glycolysis	Increased	GCs	POI, DOR, Infertility	[49]
GLUT1	Glucose Transport	Decreased	GCs	POI, DOR	[50]
GLUT3	Glucose Transport	Decreased	GCs	DOR	[17]
GLUT4	Glucose Transport	Decreased	Oocyte	POI, Infertility	[51]
Phosphoenolpyruvate	Glycolysis	Increased	GCs	DOR	[52]
G6P	PPP	Increased	GCs	DOR	[53]
NADPH	Glycolysis, OXPHOS	Decreased	GCs	DOR	[54]
NAD+	Glycolysis, OXPHOS	Decreased	GCs	DOR	[55]
ADP	Glycolysis, OXPHOS	Decreased	GCs	DOR	[17]
ATP	Glycolysis, OXPHOS	Decreased	GCs	DOR, POI, Ovarian Injury	[17, 56]
ADP/ATP	OXPHOS	Decreased	GCs	DOR	[17]
Gluconic acid	Glycolysis	Decreased	GCs	DOR	[17]
6-o-phosphono-d-gluconic acid	Glycolysis	Decreased	GCs	DOR	[57]
3-phosphoglyceric acid	Glycolysis	Decreased	GCs	DOR	[57]
Trehalose 6-phosphate	Glycolysis	Decreased	GCs	DOR	[58]
Glucose−1-phosphate	Gluconeogenesis	Decreased	GCs	DOR	[59]
α-Ketoglutarate	TCA Cycle	Decreased	GCs	POI	[60, 61]
Palmitic Acid	Glycolysis	Increased	GCs	DOR	[62]
Stearic acid	Glycolysis	Increased	GCs	DOR	[62]
Lactate	Glycolysis	Decreased	GCs	DOR	[17]
Pyruvate	Glycolysis, OXPHOS, Gluconeogenesis	Increased	GCs	POI	[63]

*Abbreviations*: *GCs* granulosa cells, *POI* premature ovarian insufficiency, *DOR* diminished ovarian reserve, *GLUT1* glucose transporter type 1, *GLUT3* glucose transporter type 3, *GLUT4* glucose transporter type 4, *PEP* phosphoenolpyruvate, *G6P* glucose−6-phosphate, *NADPH* nicotinamide adenine dinucleotide phosphate, *NAD*⁺ nicotinamide adenine dinucleotide, *ADP* adenosine diphosphate, *ATP* adenosine triphosphate, *OXPHOS* oxidative phosphorylation, *TCA cycle* tricarboxylic acid cycle

**Table 2:** Alterations in Glucose Metabolism Enzymes in Ovarian Aging

Enzyme	Metabolic Pathway Involved	Contents	Sample type	Disease Type	Reference
HK2	Glycolysis	Decreased	GCs	POI, DOR	[50]
HK3	Glycolysis	Decreased	GCs	DOR, premature ovarian failure	[64]
PKM2	Glycolysis	Decreased	GCs	POI, DOR, Infertility	[64]
PFK	Glycolysis	Decreased	GCs	DOR, Infertility	[65]
PGK1	Glycolysis	Decreased	GCs	DOR, Embryo Transfer	[66]
LDH1	Glycolysis	Decreased	GCs	DOR	[17]
Citrate Synthase	TCA Cycle	Decreased	Oocyte	DOR	[67]
PCK	Gluconeogenesis	Decreased	GCs	DOR	[17]
PDK4	Glycolysis	Decreased	GCs	Ovarian Injury	[68]
PDH	Glycolysis, TCA Cycle, OXPHOS	Decreased	Oocyte	Ovarian Dysfunction	[69, 70]
GADPH	Glycolysis	Decreased	Oocyte	Ovarian Injury	[71]
LDHA	Glycolysis	Decreased	GCs	POI	[72]
G6PD	PPP	Decreased	Oocyte	Ovarian Injury	[73]
ENO1	Glycolysis	Increased	GCs	DOR, Infertility	[74]

*Abbreviations*: *HK2* hexokinase 2, *GCs* glucocorticoids, *POI* premature ovarian insufficiency, *DOR* diminished ovarian reserve, *HK3* hexokinase 3, *PKM2* pyruvate kinase M2, *PFK* phosphofructokinase, *PGK1* phosphoglycerate kinase 1, *LDH1* lactate dehydrogenase 1, *TCA cycle* tricarboxylic acid cycle, *PCK* phosphoenolpyruvate carboxykinase, *PDK4* pyruvate dehydrogenase kinase 4, *PDH* pyruvate dehydrogenase, *OXPHOS* oxidative phosphorylation, *GAPDH* glyceraldehyde−3-phosphate dehydrogenase, *LDHA* lactate dehydrogenase A, *G6PD* glucose−6-phosphate dehydrogenase, *PPP* pentose phosphate pathway, *ENO1* enolase 1

## The regulatory role of glucose metabolism-mediated PTMs in ovarian aging

### Glycosylation

Glycosylation is one of the most prevalent PTMs in eukaryotes, endowing numerous glycoproteins with essential biological functions within the female reproductive system [75]. However, the specific roles and mechanisms of protein glycosylation in ovarian aging remain poorly understood. While the compartmentalized metabolic division of labor between oocytes and GCs is highly efficient, chronic glucose excess disrupts this balance, leading to excessive accumulation of AGEs. This process impairs mitochondrial function, induces oxidative stress, reduces glucose uptake in GCs, and ultimately accelerates ovarian aging [42]. Notably, many key proteins in the female reproductive system are glycoproteins, including follicle-stimulating hormone, luteinizing hormone, GDF-9, BMP-15, and anti-Müllerian hormone. The zona pellucida proteins ZP1, ZP2, and ZP3, which carry N-linked glycans and are highly enriched in the ovary, form the structural scaffold of the mouse zona pellucida. Their expression levels decline with ovarian aging. Recently, Wu Y. et al. established the first high-resolution N-glycoproteome landscape of the aging mouse ovary, identifying 3,746 N-glycoproteins and 5,857 N-glycosylation sites. They reported three major categories of glycosylation alterations in the aging ovary, including altered core-fucosylation, reduced LacdiNAc glycans on the zona pellucida, and increased sialylated glycans. Integrative multi-omics analysis further revealed that glycosylation primarily participates in regulating key biological pathways in the aging ovary, including antigen processing and presentation, complement cascade, ROS biosynthesis, and cell death [75]. Zhang et al. identified Cathepsin L, an oocyte-synthesized protein, as a key player. During ovarian aging, Cathepsin L undergoes aberrant N-glycosylation at asparagine 221 [76]. This form accumulates abnormally, impairs mitochondrial function, hinders autophagic-lysosomal flux, increases ROS production, and disrupts spindle assembly and chromosome segregation. These cumulative defects ultimately lead to meiotic abnormalities and compromised embryonic development. Crucially, mutating the glycosylation at asparagine 221 glycosylation site abolishes the observed detrimental effects. The expression of glycosylated Cathepsin L in the ovary gradually increases with age and is secreted into the extracellular matrix, suggesting its potential utility as a biomarker for predicting ovarian aging.

### Acetylation

In recent years, protein acetylation has emerged as a major research focus in life sciences [77–79]. Dynamically regulated by the glucose metabolic network, this post-translational modification plays a pivotal regulatory role in ovarian aging, influencing critical processes such as intercellular metabolic crosstalk, mitochondrial function, and nutrient-sensing pathways. Glucose metabolism governs acetylation through two primary, interconnected routes. Firstly, it supplies the acetyl group donor. The glycolytic end product, pyruvate, is converted to acetyl-coenzyme A in the mitochondria, which serves as the direct substrate for both histone and non-histone acetylation [80]. Fluctuations in acetyl-coenzyme A levels within ovarian cells, notably GCs and oocytes, directly modulate the acetylation status of key proteins, thereby regulating gene transcription and metabolic flux [81]. For instance, the energy metabolic reprogramming essential for oocyte maturation is dependent on acetyl-coenzyme A-mediated acetylation of specific transcription factors, such as peroxisome proliferator-activated receptor gamma coactivator 1-alpha (PGC-1α) [82]. Secondly, glucose metabolism modulates deacetylase activity. Metabolic byproducts, particularly the NAD^+^/NADH ratio, critically regulate the activity of sirtuins (SIRTs), a family of NAD^+^-dependent deacetylases that function as central energy and stress sensors [79, 83]. During ovarian aging, mitochondrial dysfunction and increased oxidative stress lead to a decline in the cellular NAD⁺ pool. This NAD⁺depletion results in the functional inhibition of key sirtuins like SIRT1 and SIRT3 [83–85]. Consequently, their downstream targets, including the anti-aging transcription factor forkhead box o3a (FOXO3a) and the mitochondrial biogenesis regulator PGC-1α, become hyperacetylated. This aberrant acetylation impairs the oocyte’s antioxidant defense, DNA repair capacity, and mitochondrial quality control, collectively accelerating its functional decline [86, 87]. Supporting this mechanism, studies have shown that aged mouse oocytes exhibit reduced SIRT1 expression, leading to hyperacetylation of p53 and subsequent apoptosis. Conversely, exogenous supplementation with NAD⁺precursors (e.g., nicotinamide mononucleotide, NMN) can partially restore SIRT1 activity, improve oocyte quality, and delay reproductive aging [88]. Therefore, interventions aimed at restoring acetylation homeostasis represent a promising strategic avenue for mitigating ovarian aging and addressing age-related infertility.

### Succinylation

Succinylation is a novel lysine post-translational modification mediated by succinyl-coenzyme A, a key metabolite derived from glucose catabolism [89]. Emerging evidence suggests that succinylation may exert more profound effects on protein structure and function than other lysine modifications, such as methylation and acetylation [18]. In ovarian aging, the glucose metabolic network primarily influences protein succinylation levels within GCs by modulating the intracellular succinyl-coenzyme A pool through both non-enzymatic and enzymatic pathways, thereby impacting folliculogenesis [90]. First, in the non-enzymatic pathway, succinyl-coenzyme A, the acyl donor for succinylation, is mainly generated via the TCA cycle from pyruvate, a product of glucose metabolism. In aged oocytes, diminished mitochondrial membrane potential and impaired ATP synthesis are often accompanied by the aberrant accumulation of TCA cycle intermediates [91]. Elevated succinyl-coenzyme A levels can lead to protein hypersuccinylation, which in turn modulates the expression of apoptosis- and senescence-associated proteins, including the downregulation of Bcl-2 and the upregulation of Caspase-3 and P21 [92]. Second, the enzymatic pathway is governed by the dynamic equilibrium between succinylation and desuccinylation. Mitochondrial GTP-specific succinyl-coenzyme A synthetase, a key enzyme in succinyl-coenzyme A metabolism, is critical for mitochondrial distribution and reproductive health in oocytes; its decreased activity is associated with delayed reproductive aging [93]. Conversely, SIRT5, the predominant mitochondrial desuccinylase, dynamically regulates the succinylation status of its target proteins in an NAD⁺-dependent manner, thereby modulating essential physiological processes such as the TCA cycle, fatty acid oxidation, and glycolysis [94]. In the aging ovary, the age-related decline in NAD⁺ levels may attenuate SIRT5’s desuccinylase activity. This results in the pathological accumulation of succinylated proteins. For instance, hypersuccinylation of mitochondrial-related factors like AIFM1 exacerbates mitochondrial fragmentation and bioenergetic failure, leading to meiotic defects and apoptosis in oocytes [90, 95]. Notably, succinylation can engage in crosstalk with other metabolism-related PTMs (e.g., lactylation), collectively fine-tuning the complex regulatory network underlying ovarian aging.

### Lactylation

Lactate, traditionally viewed as a terminal glycolytic product, is now recognized as the donor for a novel post-translational modification, protein lysine lactylation. This modification directly links cellular metabolism, particularly glycolytic flux, to epigenetic regulation, offering a new paradigm for understanding ovarian aging. Lactate can be transported into the nucleus and converted to lactyl-CoA by acyl-CoA synthetase short-chain family member 2, serving as the lactyl group donor. The dynamic level of lactylation is then regulated by “writers” such as p300 and lysine acetyltransferase 2 A, and “erasers” including SIRT1 and SIRT3, modifying both histones and non-histone proteins [96]. For instance, reduced lactate accumulation leads to decreased H3K18 lactylation, which downregulates the expression of its potential downstream target, ribosomal protein L8, a regulator of ribosomal gene transcription and cell cycle progression. This ultimately impairs meiotic competence and mitochondrial function in oocytes [97]. This aligns with prior evidence showing a positive correlation between H3K18la levels and female reproductive outcomes and embryonic development [98]. Mechanistically, alterations in lactylation levels directly impact oocyte OXPHOS, thereby influencing ROS generation and cellular quality [18]. Inflammaging, a hallmark of ovarian aging, establishes a vicious cycle: the aged ovarian microenvironment exhibits chronic low-grade inflammation and oxidative stress, which enhance glycolysis in GCs and oocytes, leading to significant lactate accumulation [99]. In GCs, this accumulated lactate, in turn, promotes apoptosis, DNA damage, and further ROS production. Notably, high lactate levels can induce PKM2 lactylation, stabilizing its dimeric form to promote glycolytic gene expression and apoptosis [100]. Therefore, therapeutic strategies targeting lactate metabolism enzymes (e.g., lactate dehydrogenase A, LDHA) or modulating the “writer/eraser” balance of lactylation present promising avenues for disrupting this detrimental cycle and improving ovarian function.

### Methylation

Methylation is the catalytic transfer of a methyl group from active methyl donors to various compounds. This process constitutes a pivotal chemical modification for proteins and nucleic acids and is intimately linked to numerous pathologies, including cancer, aging, and neurodegenerative disorders [101]. In the context of ovarian aging, the interplay between glucose metabolism and methylation, particularly histone methylation, has emerged as a critical metabolic-epigenetic regulatory axis, with elevated methylation levels strongly correlating with diminished ovarian reserve [9, 102]. In oocyte histone methylation, the aging oocyte exhibits distinct alterations in histone methylation patterns, such as reductions in H3K4me2, H3K9me3 and H4K20me2 [103]. This reprogramming is not driven by direct methyl group donation from glucose metabolites. Instead, it is driven by metabolic shifts that alter the intracellular pool and availability of universal methyl donors like S-adenosylmethionine and modulate the activity of methyltransferases and demethylases. Lysine-specific demethylase 2a, a histone demethylase that regulates glucose metabolism through modulation of H3K36me1/2/3 levels, is also involved in oocyte meiosis and embryonic development, where it plays a crucial role in epigenetic regulation [104, 105]. Beyond oocytes, methylation also exerts profound effects in GCs, where DNA methylation serves as a key epigenetic mechanism. In GCs, DNA methylation, catalyzed by DNA methyltransferases including Dnmt1, Dnmt3a, Dnmt3b, and Dnmt3l, serves as a potential predictor of aging, with altered DNMT activity and global methylation patterns being significantly associated with female infertility [104]. Supporting the metabolic connection, patients with DOR show significant upregulation of serine and glycine in GCs, amino acids crucial for one-carbon metabolism. Chronic glucose dysregulation can thereby fuel epigenetic aging in GCs by perturbing the substrate flow for DNA methylation, leading to the progressive accumulation of senescence-associated epigenetic marks [106]. Experimental evidence further underscores this link: reduced DNMT activity correlates with declining fertility in POI model mice, while knockdown of the demethylase fat mass and obesity-associated protein exacerbates pro-senescent phenotypes in GCs [107]. Critically, altered DNA methylation within the promoter regions of steroidogenic genes in GCs suppresses hormone secretion and accelerates follicular atresia. Collectively, these findings delineate a mechanism whereby glucose metabolism orchestrates ovarian aging by governing the substrate supply, enzymatic activity, and signaling networks that underpin the epigenetic landscape of methylation.

### Phosphorylation

**Fig. 3:**
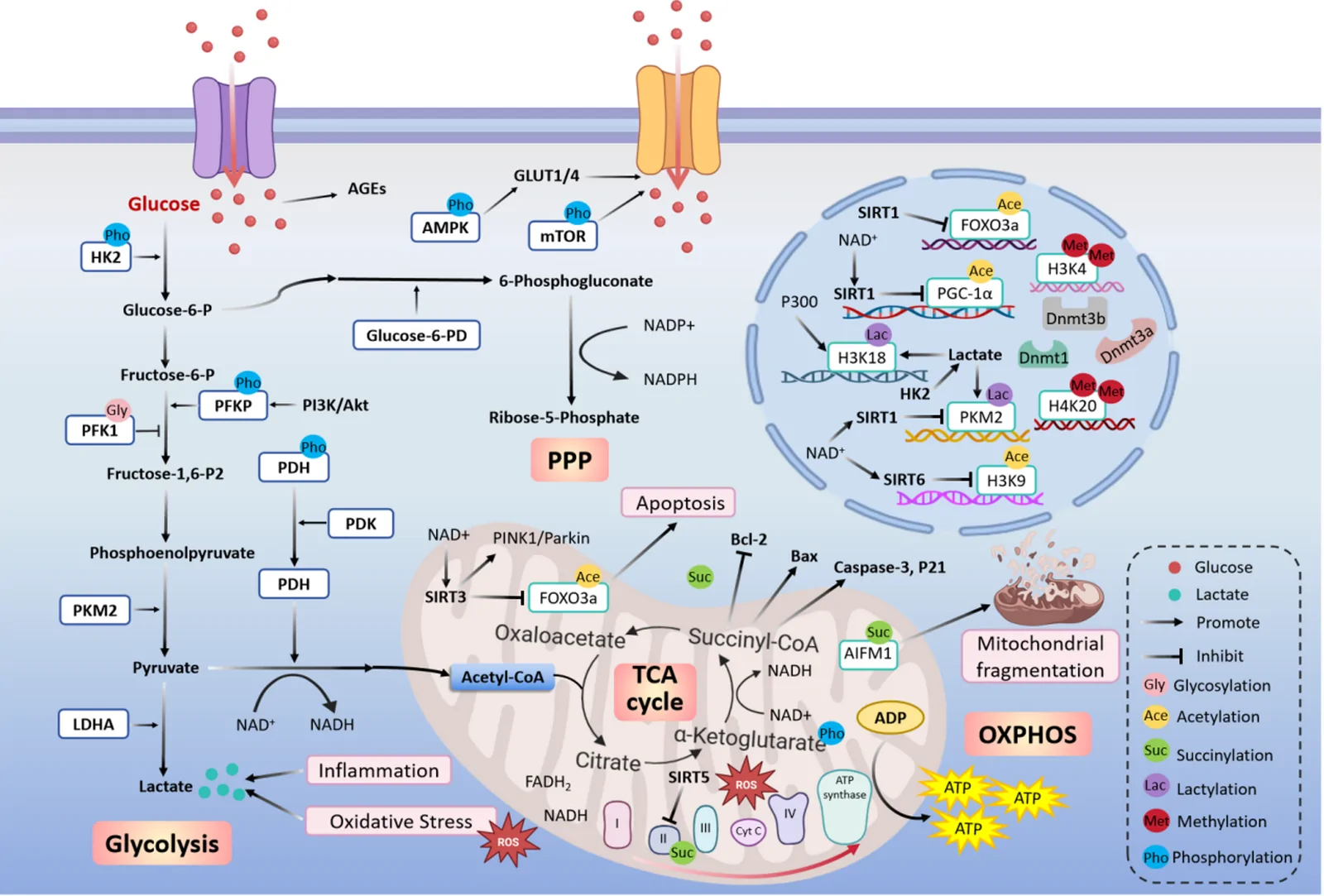
Schematic diagram illustrating ovarian glucose metabolism and associated PTMs. Glycolysis Pathway: Glucose is phosphorylated by HK2 to glucose-6-phosphate, which is isomerized to fructose-6-phosphate. Fructose-6-phosphate is then catalyzed by phosphofructokinase-1 or its platelet-type isoform (PFKP) to fructose-1,6-P, eventually yielding phosphoenolpyruvate. Phosphoenolpyruvate is converted to PKM2, and finally reduced to lactate by LDHA. PPP: glucose-6-phosphate is oxidized by G6PD to 6-phosphogluconate, which is further decarboxylated to generate ribose-5-phosphate and NADPH. TCA Cycle: Pyruvate enters the mitochondria and is converted to acetyl-coenzyme A by the PDH. acetyl-coenzyme A then enters the TCA cycle, producing key metabolites such as α-ketoglutarate and succinyl-coenzyme A, along with reducing equivalents (NADH, FADH_2_). OXPHOS: NADH and FADH₂ derived from the TCA cycle donate electrons to the mitochondrial electron transport chain (complexes I–IV), establishing a proton gradient that drives ATP synthesis via ATP synthase. PTMs in Metabolic Regulation: Glycosylation: Elevated glucose levels promote the formation of AGEs. O-GlcNAcylation of phosphofructokinase-1, for example, downregulates its enzymatic activity. Acetylation: SIRT1 deacetylates PGC-1α and FOXO3a, while SIRT3-mediated deacetylation of FOXO3a modulates apoptosis. SIRT6 deacetylates histone H3K9 to enhance genomic stability. Succinylation: Driven by succinyl-coenzyme A, hyper-succinylation can occur under metabolic stress. SIRT5 acts as the major desuccinylase and participates in apoptosis regulation. Lactylation: Lactate serves as a substrate for lactylation, modifying both histone (e.g., H3K18) and non-histone proteins such as PKM2. Methylation: Dynamically regulated by methyltransferases and demethylases, histone methylation is closely linked to cellular life-cycle processes. Phosphorylation: AMPK and mTOR signaling pathways promote glucose uptake. Phosphorylation enhances the activity of HK2 and PFKP, while inhibiting PDH activity. The figure was created using BioRender. Abbreviations: GCs, granulosa cells; DOR, diminished ovarian reserve; POI, premature ovarian insufficiency; OXPHOS, oxidative phosphorylation; TCA, tricarboxylic acid; PPP, pentose phosphate pathway; PTMs, post-translational modifications; AGEs, advanced glycation end products; HK2, hexokinase 2; PKM2, pyruvate kinase M2; LDHA, lactate dehydrogenase A; PFKP, platelet-type PFK; G6PD, glucose-6-phosphate dehydrogenase; PDH, pyruvate dehydrogenase complex; PGC-1α, peroxisome proliferator-activated receptor gamma coactivator 1-alpha; FOXO3a, forkhead box O3a; AMPK, AMP-activated protein kinase; mTOR, mechanistic target of rapamycin; NMN, nicotinamide mononucleotide

Phosphorylation serves as a central signaling nexus in ovarian aging, orchestrating a tightly interwoven regulatory network that directly drives glucose metabolism while, in turn, being extensively triggered by its metabolic intermediates [108–110]. At the signaling level, phosphorylation dynamically regulates the activity of core nutrient-sensing pathways, including AMP-activated protein kinase (AMPK) and the mechanistic target of rapamycin (mTOR), thereby integrating metabolic state with cellular senescence and reproductive function [111]. AMPK, the cellular “energy switch,” is activated by phosphorylation in response to an increased AMP/ATP ratio, a direct readout of glucose metabolism. Phosphorylated AMPK promotes the membrane translocation of GLUT1/4, activates phosphofructokinase-1, and inhibits glycogen synthase, coordinately enhancing glucose uptake and utilization to bolster pyruvate synthesis and ATP production. In GCs from patients with DOR, a significant downregulation of AMPK transcriptional levels is observed, contributing to glucose-energy metabolism impairment and reduced glucose uptake [17]. Conversely, the mTOR pathway, particularly mTOR complex 1, promotes energy synthesis by phosphorylating downstream targets like ribosomal protein S6 kinase 1 to induce the expression of glycolytic genes such as GLUT1, HK2, and PKM2. While mTOR complex 2 primarily modulates insulin signaling to influence glucose metabolism, studies indicate that mTOR signaling is aberrantly and specifically hyperactivated in the aging ovary. This hyperactivation can trigger apoptotic cascades in GCs, promote excessive primordial follicle activation, and induce the release of the senescence-associated secretory phenotype, collectively accelerating ovarian functional decline [112]. Beyond these signaling pathways, phosphorylation events directly governing oocyte meiosis are also disrupted during ovarian aging. The phosphorylation status of cyclin-dependent kinase 1 serves as the master switch driving germinal vesicle breakdown and spindle assembly, while the MAPK/ERK phosphorylation cascade regulates cumulus cell expansion and ovulation-related gene expression. In aged oocytes, the dynamic balance of these phosphorylation events is disrupted, manifesting as aberrant cyclin-dependent kinase 1 dephosphorylation and reduced MAPK activity, which directly contribute to meiotic arrest and chromosome misalignment [113, 114]. Thus, phosphorylation operates as a multi-layered and dynamic master regulator, integrating metabolic and signaling networks to exert a profound influence on the progression of ovarian aging (Fig. 3).

## Crosstalk between glucose metabolic homeostasis and ovarian dysfunction

**Fig. 4:**
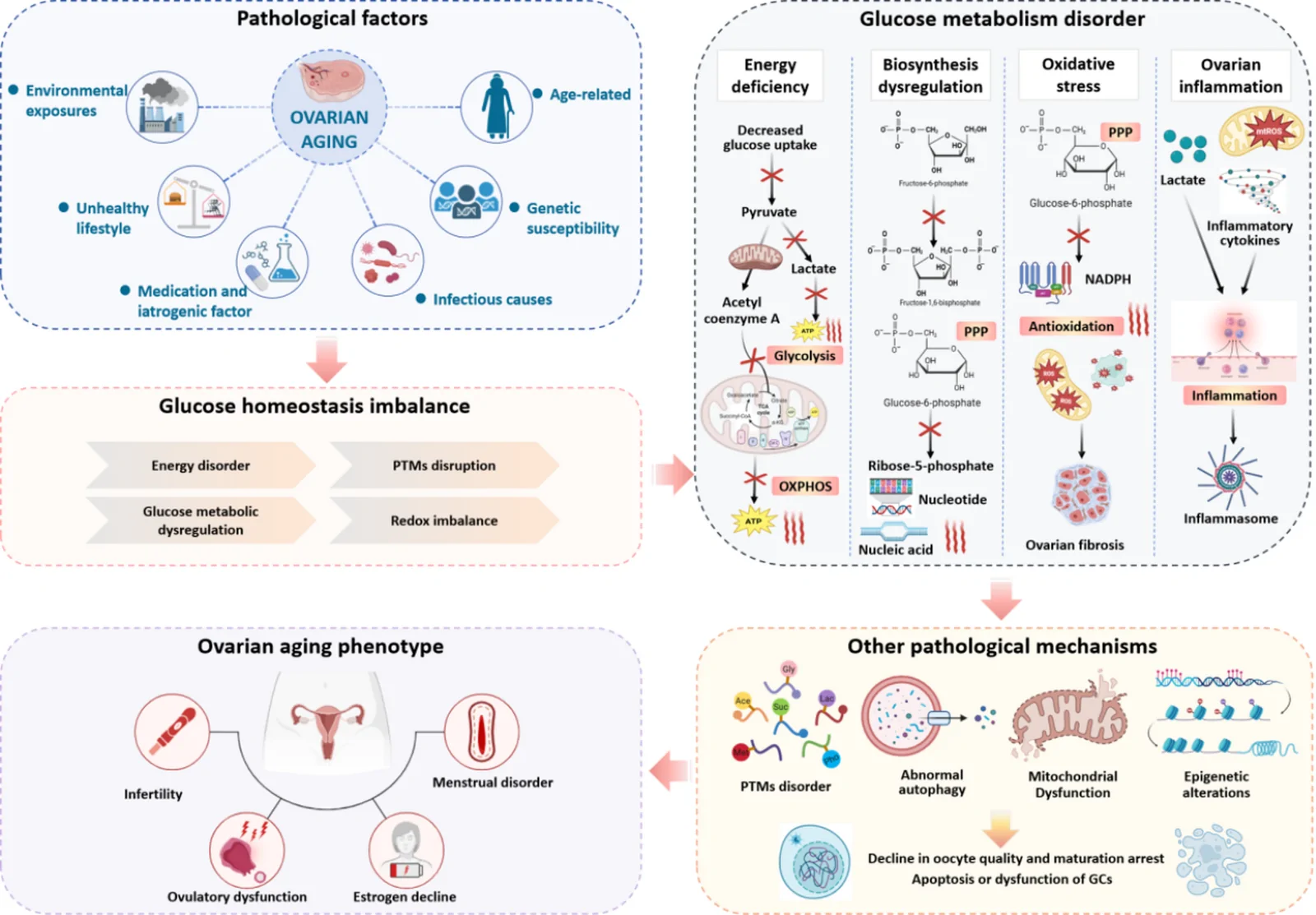
Pathological factors, glucose metabolic reprogramming, and clinical phenotypes of ovarian aging. Ovarian aging is driven by multiple pathological factors, including environmental exposures, unhealthy lifestyle, iatrogenic factors, and genetic factors. These factors collectively induce glucose metabolism disorders, which are characterized by energy deficiency and biosynthesis dysregulation, along with oxidative stress and chronic inflammation. These alterations further promote abnormalities in protein PTMs, autophagy, mitochondrial dysfunction, and epigenetic alterations. Consequently, these metabolic abnormalities lead to a decline in oocyte quality and maturation arrest, as well as apoptosis or dysfunction of GCs. The resulting clinical manifestations include infertility, ovulatory dysfunction, estrogen decline, and menstrual irregularities. Abbreviation: ATP, adenosine triphosphate; OXPHOS, oxidative phosphorylation; PPP, pentose phosphate pathway; NADPH, nicotinamide adenine dinucleotide phosphate; ROS, reactive oxygen species; mtROS, mitochondrial reactive oxygen species; PTMs, post-translational modifications; Gly, Glycosylation; Ace, Acetylation; Suc, Succinylation; Lac, Lactylation; Met, Methylation; Pho, Phosphorylation; GCs, granulosa cells. The figure was created using BioRender

A hallmark of female reproductive aging is the marked decline in both the quantity and quality of oocytes, characterized by a pronounced increase in chromosomal abnormalities. This decline underlies the clinical challenges faced by advanced maternal age patients, including low success rates in in vitro fertilization, elevated miscarriage rates, and a higher incidence of birth defects in assisted reproductive technologies [115]. The intricate interplay between disrupted glucose metabolic homeostasis and ovarian dysfunction is mediated by several interconnected pathological mechanisms, including oxidative stress, mitochondrial dysfunction, chronic inflammation, and dysregulated autophagy. These mechanisms collectively form a self-reinforcing network that accelerates functional decline (Fig. 4).

### Oxidative stress

Organisms rely on a sophisticated redox system to control reactive ROS and maintain oxidative homeostasis. Oxidative stress denotes an imbalance in this system. At physiological levels, oxidative stress can activate cytoprotective signaling, induce coordinated mitochondrial dynamics (fusion/fission), and promote selective autophagy, thereby clearing dysfunctional cells. However, pathological oxidative stress inflicts severe cellular damage and aberrant apoptosis, profoundly impairing folliculogenesis and ovulation, and ultimately leading to ovarian dysfunction. With advancing age, dysregulated glucose metabolism promotes the aberrant accumulation of ROS within the ovary, causing oxidative damage, exacerbating oxidative stress, and inducing cellular dysfunction and death. Furthermore, oxidative stress reciprocally impairs metabolic coupling between GCs and the oocyte by hindering processes like pyruvate transport, leading to oocyte maturation arrest. Research has identified harmful mutations and downregulation of the oxidative stress-responsive gene nuclear receptor coactivator 7 in women with POI or physiological ovarian aging. Silencing nuclear receptor coactivator 7 recapitulates an aging phenotype, marked by reduced fertility, decreased estradiol production, increased follicular atresia, and ovarian fibrosis [56]. Concurrently, Li et al. demonstrated that the accumulation of AGEs in GCs within the ovarian microenvironment exacerbates oxidative stress, damages mitochondrial function, and inhibits mitophagy. Silencing SIRT3 or PINK1 further aggravates these detrimental effects [116]. Conversely, upregulating SIRT3 delays ovarian aging and restores mitochondrial function by promoting mitophagy, while also enhancing estradiol and progesterone synthesis to maintain ovarian physiology. The sirtuin family, particularly SIRT1 and SIRT3, serves as a critical nexus linking glucose metabolism to oxidative stress regulation and nutrient sensing [116]. SIRT1 modulates key stress-response proteins like FOXO, upregulates antioxidant enzymes, and mediates PGC-1α to promote mitochondrial biogenesis and OXPHOS. Its deficiency in oocytes or GCs directly compromises cell quality, steroidogenesis, and pregnancy outcomes [117]. Other sirtuin isoforms also contribute to mitigating ovarian aging: SIRT2 deacetylates transcription factors (e.g., FOXO, p53, NF-κB) to prevent oxidative stress damage [118]; SIRT5 activates NADPH-producing enzymes to bolster antioxidant defense [54]; and SIRT6 safeguards against oxidative stress-induced DNA damage to preserve ovarian function [119].

### Mitochondrial dysfunction

Mitochondria, serving as the metabolic command centers of the cell, generate the primary energy required for oocyte maturation and early embryonic development through OXPHOS [7]. Oocytes are particularly rich in mitochondria, which are crucial for supporting the dynamics of spindle assembly and ensuring accurate chromosome segregation. Mounting evidence identifies mitochondrial dysfunction as a central hallmark of ovarian aging, manifesting as mitochondrial DNA damage, imbalanced dynamics, increased oxidative stress, impaired biogenesis, and diminished membrane potential. Mechanistically, mitochondrial function is regulated at multiple levels. SIRT2 promotes mitochondrial biogenesis and modulates the fusion/fission cycle to sustain ATP production, thereby supporting oocyte meiosis. Mitochondrial Rho GTPase 1 enhances the activity of the electron transport chain and the TCA cycle to meet the heightened energy demands of oocyte maturation. Supporting the clinical relevance, ATP levels within GCs from young women are approximately 4.3 times higher than those from older women, underscoring a direct link between compromised energy output and diminished reproductive potential [120]. Exogenous supplementation with GDF-9 can elevate mitochondrial activity and increase the proportion of metabolically active mitochondria, promoting oocyte maturation [121]. Meanwhile, the DNA-binding inhibitor 3 optimizes mitochondrial morphology and function by regulating the expression of key proteins involved in mitophagy (Parkin) and dynamics (Dynamin-related protein 1, Mfn1/2), thereby supporting steroidogenesis. Critically, glucose metabolic homeostasis is tightly coupled to mitochondrial integrity. Metabolic dysregulation reduces mitochondrial membrane potential and electron transport chain efficiency, leading to ROS accumulation and accelerating GCs senescence and apoptosis. Growth differentiation factor 15 (GDF-15), a key plasma biomarker of mitochondrial stress, integrates mitochondrial, metabolic, and immune signals. Its elevated levels are significantly correlated with diminished ovarian reserve, as indicated by a reduced anti-Müllerian hormone to follicle-stimulating hormone ratio [122]. Furthermore, the SIRT1/PGC-1α signaling axis, a master regulator in glucose metabolism, promotes mitochondrial biogenesis via deacetylation. The age-related decline in this pathway, leading to a reduction in mitochondrial mass, constitutes a primary cause of reproductive decline. Resveratrol, a SIRT1 agonist, can rescue this defect by increasing mitochondrial quantity and improving reproductive outcomes [123]. These findings collectively elucidate the pivotal role of mitochondria in ovarian aging and provide a theoretical foundation for developing interventions targeting the metabolic-mitochondrial axis to counteract reproductive decline.

### Chronic inflammation

Chronic inflammation stands as a hallmark pathological feature of ovarian aging, actively driving follicular depletion and functional decline through the dysregulation of the immune system and key cellular signaling pathways. This inflammatory process ultimately culminates in reproductive impairment. Substantial evidence establishes a strong link between inflammation and conditions such as POI. The nucleotide-binding oligomerization domain, leucine-rich repeat and pyrin domain containing 3 (NLRP3) inflammasome and NF-κB pathways have been identified as the central inflammatory axes mediating age-related fertility decline. Notably, follicular fluid from POI patients and aged ovaries exhibits significantly elevated levels of pro-inflammatory cytokines (e.g., IL-1β, IL-18, IL-6) and ROS, which inversely correlate with ovarian reserve metrics [124]. Interventions aimed at mitigating inflammation have been shown to improve reproductive endocrine profiles and ovarian reserve function. In this pathological context, glucose metabolism and inflammation engage in a bidirectional, self-reinforcing regulatory loop. On one hand, dysregulated glucose metabolism directly instigates and exacerbates inflammation. For instance, immune cells like M1 macrophages and neutrophils undergo metabolic reprogramming in inflammatory milieus, preferentially utilizing glycolysis. The resulting metabolic intermediates fuel cytokine production (e.g., IL-1β, tumor necrosis factor-alpha) and stabilize inflammatory transcription factors like hypoxia-inducible factor 1α. A chronically hyperglycemic environment promotes ROS accumulation and NF-κB pathway activation, leading to mitochondrial damage and apoptosis [125]. Moreover, AGEs can impair the insulin-mediated PI3K/Akt signaling pathway, a master regulator of primordial follicle growth, by blocking GLUT4 membrane translocation and inducing insulin resistance, disruptions directly linked to infertility and POI. On the other hand, inflammation reciprocally induces glucose metabolic dysfunction and promotes apoptosis. In models of DOR, the upregulation of hypoxia-inducible factor 1α directly induces the overexpression of key glycolytic proteins (GLUT1, HK2, LDHA), forcing a metabolic shift towards glycolysis and disrupting cellular energy homeostasis [126]. Inhibiting the NLRP3 inflammasome in GCs can increase the expression of rate-limiting glycolytic enzymes, significantly suppress apoptosis, and ultimately improve endocrine function [127]. Critically, inflammation itself is a potent trigger of apoptosis. Ovarian tissues from aged rats (e.g., 60weeks) demonstrate a significant positive correlation between heightened inflammatory markers and increased GCs apoptosis [128]. Mechanistic studies further show that atorvastatin can ameliorate ovarian dysfunction by activating the PI3K/Akt pathway, downregulating the pro-apoptotic protein Bax, and upregulating the anti-apoptotic protein Bcl-2. Therefore, chronic inflammation and glucose metabolic dysregulation in ovarian aging are not merely concurrent events but are locked in a vicious, self-perpetuating cycle of “inflammation-metabolic reprogramming,” which synergistically drives ovarian functional decline.

### Autophagy

Autophagy is a highly conserved intracellular degradation process, regulated by autophagy-related genes, which delivers damaged organelles and misfolded proteins to lysosomes for clearance [129]. It encompasses three primary forms: macroautophagy, microautophagy, and chaperone-mediated autophagy. Within the ovary, autophagy serves as a pivotal regulator of follicular development, oocyte maturation, and GCs function. Moderate autophagy acts as a quality control mechanism, clearing harmful byproducts of oxidative stress and mitochondrial dysfunction [130, 131]. However, both its excessive activation and functional impairment can lead to cellular energy depletion and the initiation of programmed cell death, thereby driving ovarian aging. Specifically, defective mitophagy, the selective autophagic removal of mitochondria, is strongly implicated in ovarian functional decline. Crucially, autophagy is not a passive response but an active biological process triggered through a cascade of intracellular signaling upon sensing extracellular and intracellular cues. Key enzymes and intermediates of glucose metabolism are central regulators of autophagy. For example, pyruvate dehydrogenase kinase 4 (PDK4) induces autophagy by upregulating the expression of the core autophagy protein Beclin-1 and downregulating p62, while simultaneously suppressing glycolysis by inhibiting GLUT1 and HK2 expression [132]. PKM2, beyond its canonical role in the terminal step of glycolysis, functions as a protein kinase and transcriptional co-activator, influencing processes such as cell proliferation, apoptosis, and migration. PKM2 interacts with the Akt/mTOR signaling pathway, a primary negative regulator of autophagy, where mTOR serves as the master inhibitory kinase [133]. Studies have shown that SIRT1 inhibitors can suppress PKM2-mediated cancer cell metabolism via the PI3K/Akt/mTOR pathway and induce autophagic cell death. Under glucose-deprived conditions, HK2 can bind to and sequester mTOR, causing its dissociation from the lysosome and thereby inducing autophagy [134]. The hypoxic ovarian microenvironment presents a critical scenario linking energy metabolism to autophagy. In this setting, GCs primarily rely on glycolysis to meet their energetic demands, leading to lactate accumulation and the upregulation of glycolytic proteins. Research indicates that lactate promotes the stabilization of hypoxia-inducible factor 1α, which can disrupt the Beclin-1/Bcl-2 complex to initiate autophagy or activate the PINK1/Parkin pathway to induce mitophagy, ultimately suppressing aberrant GC apoptosis [135]. Accumulating evidence highlights lactate-induced protein lactylation as a key mechanistic link between glycolysis and autophagy. For instance, inhibiting AMPK lactylation has been shown to effectively promote autophagy and delay cellular senescence. Furthermore, lactylation can stabilize Transcription Factor EB, a master regulator of autophagy and lysosomal gene expression, to enhance autophagic flux [136]. However, the precise role and regulatory mechanisms of lactylation in modulating autophagy under hypoxic conditions remain incompletely understood. Future research is warranted to elucidate the function of histone and non-histone lactylation in GC autophagy, which will provide deeper insights into how glucose metabolism-mediated PTMs govern the critical pathways of ovarian aging.

## Potential intervention strategies for restoring glucose metabolic homeostasis to attenuate ovarian aging

The recognition that dysregulation of glucose metabolic homeostasis constitutes a central pathological feature of ovarian aging offers a novel therapeutic paradigm, moving beyond traditional hormone replacement strategies. Consequently, restoring this metabolic equilibrium emerges as a promising and innovative intervention avenue. The following section discusses therapeutic strategies targeting distinct nodes of the glucose metabolic network. These include pharmacological interventions, as well as cell- and exosome-based therapies (Table 3). Pharmacological approaches primarily aim to modulate glucose uptake and utilization, target specific metabolic pathways, and correct aberrant metabolite-driven PTMs. Collectively, these interventions are designed to re-establish glucose metabolic homeostasis, with the ultimate goal of delaying or even reversing the trajectory of ovarian aging.

**Table 3:** Therapeutic Strategies Targeting Glucose Metabolic Homeostasis in Ovarian Aging: Mechanisms and Interventions

Category	Evidence Level	Drug/Cell	Dosage	Model	Age	Sample size (*n*)	Disease	Regulatory mechanism(s)	Reference
Pharmacological Intervention									
Modulation of Glucose Uptake and Utilization	Human case	Empagliflozin	10 mg/day	Human	32 years old	1	POI and Glycogen storage disease type 1b	Empagliflozin might be associated with reduced oxidative stress and inflammatory markers, enhanced ovarian angiogenesis, restored menstrual cyclicity, and modulated follicle-stimulating hormone and luteinizing hormone levels, potentially facilitating natural conception, although large-scale studies are still needed for further validation.	[137]
	Animal	Exenatide	10 µg/kg/day	Rats	Not reported	24	Type 2 Diabetes Mellitus with Ovarian Injury	Exenatide activates the GLP-1 receptor to promote insulin secretion, reduce hepatic glucose output, and enhance glucose uptake and utilization in adipocytes and muscle cells. This process concurrently alleviates oxidative stress, ultimately ameliorating endometrial and ovarian tissue fibrosis and improving ovarian reserve function.	[138]
	Human trial	Hishi	100 mg/day	Human	38–64 years old	64	Advanced-age patients undergoing assisted reproductive technology	Hishi reduces AGEs in serum and follicular fluid, improves oocyte quality and endometrial receptivity, and increases the live birth rate in advanced-age patients.	[139]
	Cell	Urolithin A	20µmol/L	Human GCs	≤ 35 years old	477	Infertility	Urolithin A activates GCs mitophagy, downregulates p62, and upregulates the expression of PINK1, Parkin, and LC3-II proteins, thereby improving mitochondrial function and promoting the synthesis of estradiol and progesterone. This beneficial process is effectively counteracted against the pathological background induced by AGEs.	[116]
Targeting the Glucose Metabolic Network	Animal	PQQ	10 mg/kg/day	Rats	8 weeks	30	POI	PQQ upregulates the expression of HK2, PKM2, and LDHA in GCs, elevates lactate concentration, and inhibits the NLRP3 inflammasome to alleviate inflammatory responses, thereby restoring ovarian reserve function.	[127]
	Animal	Alpha-ketoglutarate	250 mg/kg/day	Rats	10 weeks	30	POI	Alpha-ketoglutarate downregulates the pro-apoptotic proteins Bax and Caspase−3 while upregulating the anti-apoptotic protein Bcl−2, thereby inhibiting apoptosis in GCs. Concurrently, it upregulates the expression of key glycolytic enzymes, including HK2, PKM2, and LDHA. This enzymatic reprogramming increases cellular levels of ATP and D-FBP, elevates lactate concentration, reduces pyruvate accumulation, and ultimately enhances glycolytic flux.	[60]
	Cell		100µmmol/L	Bovine	Not reported	200–240	Normal	Alpha-ketoglutarate modulates the PPP, the TCA cycle, and overall glucose metabolism in oocytes. It increases the levels of fructose−6-phosphate, 3-phosphoglycerate, and 2-phosphoglycerate, while concurrently reducing phosphoenolpyruvate, fructose−1,6-bisphosphate, gluconolactone, and gluconate. This metabolic reprogramming in GCs provides crucial support for oocyte maturation, ultimately enhancing the blastocyst formation rate.	[140]
	Animal	Kuntai capsule	0.4 mg/kg/day, 0.8 mg/kg/day, 1.6 mg/kg/day	Mice	6–8 weeks	40	POI	Kuntai Capsule upregulates the expression of SIRT5 and FOXO3a proteins and enhances the activity of antioxidant enzymes, such as manganese superoxide dismutase. This action protects mitochondria from oxidative damage, augments OXPHOS, and ameliorates the condition of POI.	[141]
	Human trial	Metformin	200 mg/day	Human	32.2 ± 2.8 years old	60	POI	Metformin downregulates leptin and follicle-stimulating hormone levels while upregulating estradiol concentration. It enhances AMPK phosphorylation, alleviates inflammation, and restores cellular bioenergetic pathways to maintain energy homeostasis.	[142]
	Animal	Curcumin	100 mg/kg/day	Mice	3 weeks	200	Aged	In the aged model, curcumin treatment promoted the expression of GDF-9, BMP-15, and glutathione, reduced ROS levels, and improved glucose metabolism. Ultimately, these effects enhanced fertilization and blastocyst formation rates.	[143]
	Animal	Quercetin	100 mg/kg/day	Rats	8 weeks	30	POI	Quercetin mitigates ovarian oxidative stress, inhibits cell apoptosis, and reduces follicular atresia by downregulating PARP1 expression and suppressing GSK-3β activity.	[144]
Modulating metabolite-driven PTMs	Cell	Sodium lactate	10mmol/L	Mice	10 weeks	Not reported	Normal	Sodium lactate increased the lactylation levels of H3K14, H4K12, and H4K5, thereby activating genes beneficial for oocyte maturation and development. These changes ultimately enhanced embryonic development outcomes. KEGG pathway analysis further revealed that differentially expressed genes were primarily enriched in pathways related to OXPHOS, folate biosynthesis, and glycolysis. Notably, alterations in lactylation levels were shown to affect OXPHOS activity in oocytes.	[145]
	Cell	Melatonin	1 µg/mL	three-way hybrid piglets GCs [(Duroc × Landrace) × Large White]	5 weeks	12	Ovary injury	Melatonin ameliorates ovarian function by upregulating H3K18 lactylation in GCs, which subsequently enhances lactate accumulation, promotes glycolysis, and suppresses ferroptosis and oxidative stress.	[146]
	Animal		20 mg/kg/day	Mice	8 weeks	18			
	Cell	Resveratrol	50µmol/L	Rat GCs	3 weeks	1 × 10^4^ cells/dish	POI	Resveratrol pretreatment can inhibit glucocorticoid-induced oxidative stress, downregulate the autophagy marker LC3B, increase SIRT1 expression, reduce levels of Beclin-1, Bax, and Caspase-3, enhance mitochondrial OXPHOS, and suppress cell apoptosis.	[147]
	Animal	Coenzyme Q10	22 mg/kg/day	Mice	9 months	21	Aged	Coenzyme Q10 significantly improved fertility in aged mice, increasing follicle count, ovulation rate, and litter size. It also enhanced mitochondrial respiratory rate, membrane potential, reduced reactive oxygen species levels, and improved spindle morphology in oocytes. Furthermore, TCA cycle activity was elevated, accompanied by increased ATP production.	[148]
	Animal	Melatonin combined with resveratrol	Melatonin 20 mg/kg/day and resveratrol 10 mg/kg/day	Rats	8 weeks	42	POI	The treatment combining melatonin with resveratrol upregulates SIRT1 expression, deacetylates the downstream target FOXO3a, promotes expression of the anti-apoptotic protein Bcl2, inhibits fibrotic factors and oxidative stress, and ultimately alleviates ovarian fibrosis.	[149]
Cell and Exosome Therapy									
	Animal	Human placenta-derived mesenchymal stem cells	2 × 10^6^ naïve human placenta-derived mesenchymal stem cells	Rats	8 weeks	Not reported	Ovarian injury	human placenta-derived mesenchymal stem cells activated the AMPK pathway, upregulating the expression of GLUT4 and insulin-like growth factor-binding protein 2, which in turn promoted glucose metabolism and improved mitochondrial function, resulting in increased ATP content and mitochondrial DNA copy number.	[150]
	Animal	human UC-mesenchymal stem cell-derived exosomes	1 × 10^10^ particles human UC-mesenchymal stem cell-derived exosomes	Rats	8 weeks	36	POI	human UC-mesenchymal stem cells-derived exosomes increased serum levels of estradiol and anti-Müllerian hormone, elevated the number of follicles at various developmental stages, improved ovarian function, and enhanced live birth rates. Additionally, they were involved in regulating cellular senescence, apoptosis, glucose metabolism pathways, ATP synthesis, and inflammatory status.	[151]
	Animal	peripheral blood mononuclear cell transplantation combined with platelet-rich plasma	4 × 10^6^ peripheral blood mononuclear cells combined with 4 × 10^7^ platelet-rich plasma	Rats	13 months	72	Aged	The combination of PBMC and platelet-rich plasma restored the estrous cycle, increased serum levels of anti-Müllerian hormone and estradiol, and augmented follicle numbers. This intervention also improved embryo implantation and live birth rates, while significantly promoting follicular angiogenesis and upregulating the expression of vascular endothelial growth factor, platelet endothelial. cell adhesion molecule-1, and α-smooth muscle actin. Moreover, PBMC + platelet-rich plasma co-treatment elevated the expression of HK, PFK, PKM2, SIRT1, and LDHA in GCs. These effects were mechanistically linked to the enhancement of glucose metabolism.	[152]

“Not reported” indicates that the information (e.g., age or sample size) was not reported in the original publication and could not be retrieved. “Cell” denotes in vitro experiments and is not a substitute for clinical therapeutic application.

*Abbreviations*: *POI* premature ovarian insufficiency, *GLP-1* glucagon-like peptide-1, *AGEs* advanced glycation end products, *GCs* granulosa cells, *LC3-II* microtubule-associated protein 1 light chain 3 beta (LC3-II), *PQQ* pyrroloquinoline quinone, *HK2* hexokinase 2, *PKM2* pyruvate kinase M2, *LDHA* lactate dehydrogenase A, *NLRP3* NLR family pyrin domain containing 3, *Bcl-2* B-cell lymphoma 2, *Bax* Bcl-2-associated X protein, *ATP* adenosine triphosphate, *PPP* pentose phosphate pathway, *TCA cycle* tricarboxylic acid cycle, *SIRT5* sirtuin 5, *FOXO3a* forkhead box O3a, *OXPHOS* oxidative phosphorylation, *GDF-9* growth differentiation factor 9, *BMP-15* bone morphogenetic protein 15, *GSK-3β* glycogen synthase kinase-3 beta, *H3K14* histone H3 lysine 14, *H4K12* histone H4 lysine 12, *H4K5* histone H4 lysine 5, *KEGG* Kyoto Encyclopedia of Genes and Genomes, *H3K18* histone H3 lysine 18, *SIRT1* sirtuin 1, *GLUT4* glucose transporter type 4, *DNA* deoxyribonucleic acid, *PBMC* peripheral blood mononuclear cell, *HK* hexokinase, *PFK* phosphofructokinase

### Pharmacological interventions

#### Modulating glucose uptake and utilization

Restoring glucose metabolic homeostasis in ovarian aging necessitates multi-faceted intervention strategies, including precise modulation of glucose uptake and utilization. In patients with DOR, GCs exhibit significantly reduced glucose content and downregulated expression of GLUT1 and GLUT3 compared to those with normal ovarian reserve [17]. Notably, follicle-stimulating hormone levels positively correlate with fasting serum glucose concentrations, and elevated serum glucose is also observed in patients with POI [153, 154]. However, the relationship between intra-ovarian glucose levels and function is not linear but follows an inverted U-shaped curve. While deficiency impairs function, a chronic hyperglycemic environment promotes the accumulation of AGEs, induces oxidative stress, and exacerbates ovarian damage. This complex relationship shifts the therapeutic focus toward the precise regulation of glucose uptake and utilization. In conditions like POI and DOR, enhancing cellular glucose utilization or facilitating the clearance of deleterious AGEs is crucial for improving the function of oocytes and their surrounding somatic cells. As reported in a single case report by Koloutsou ME et al. [137], glucose transport modulators, including the sodium-glucose cotransporter 2 inhibitor empagliflozin, not only help lower systemic glucose levels but also reduce oxidative stress and exert anti-inflammatory effects. These changes, in turn, are linked to improved reproductive outcomes. The glucagon-like peptide-1 analog exenatide activates the GLP-1 receptor to promote insulin secretion, reduce hepatic glucose output, and enhance glucose uptake and utilization in adipocytes and muscle cells [138]. This action is associated with reduced endometrial and ovarian tissue fibrosis and improved markers of ovarian reserve. Regarding AGEs clearance, compounds like Hishi have been shown to lower AGEs levels in serum and follicular fluid of advanced-age patients, correlating with increased pregnancy and live birth rates [139]. However, it must be acknowledged that this finding is limited to a small sample size of 64 cases and has not yet been clinically validated in large-scale studies. Additionally, long-term safety assessments of Hishi have not been conducted, and the study excluded patients with severely diminished ovarian reserve; thus, its efficacy of Hishi in this patient population remains to be validated. Furthermore, urolithin A can activate mitophagy, which is suppressed by AGEs in GCs of infertile patients, and promote steroid hormone synthesis in GCs [116].

#### Targeting the glucose metabolic network

The glucose metabolic network is primarily constituted by three interconnected pathways: OXPHOS, the PPP, and glycolysis. Restoring the dynamic equilibrium within this network represents a promising strategy to delay reproductive aging [18]. OXPHOS serves as the primary energy-generating process in oocytes, relying on pyruvate derived from glycolysis as a key substrate. The PPP is crucial for oocyte maturation, although its precise mechanistic role remains to be fully elucidated. This may stem from the distinct metabolic compartmentalization: GCs are highly glycolytic, whereas oocytes have limited direct glucose utilization and instead acquire critical metabolites like NADPH from GCs and follicular fluid to maintain redox homeostasis. The aging process disrupts this intricate cooperative network. Rate-limiting enzymes of glycolysis act as pivotal nodes at the crossroads of these pathways, directly dictating the metabolic fate of glucose. Therefore, modulating the activity of key metabolic enzymes, such as HK2, PKM2, and LDHA, can restore metabolic balance [155–157]. For instance, in GCs, pyrroloquinoline quinone (PQQ) upregulates the expression of HK2, PKM2, and LDHA, increases lactate concentration, and inhibits the NLRP3 inflammasome to alleviate inflammation, collectively supporting ovarian reserve function. However, whether PQQ directly affects oocytes remains unclear, and this represents a critical knowledge gap that warrants further investigation [127]. Similarly, alpha-ketoglutarate can modulate the expression of these enzymes and regulate the PPP, glycolysis, and the TCA cycle, thereby inhibiting apoptosis, elevating lactate levels, promoting oocyte maturation, and improving blastocyst formation rates [60]. The herbal formulation Kuntai capsule has been shown to upregulate the FOXO3a/SIRT5 signaling axis in mice, thereby regulating both OXPHOS and inflammatory pathways in the treatment of POI [141]. A small-sample clinical observational study found that metformin mediates the phosphorylation of the cellular energy sensor AMPK, upregulates cellular energy metabolism pathways, and improves ovarian function [142]. Notably, oocyte-secreted factors GDF-9 and BMP-15 also contribute to metabolic regulation by modulating the activity of key enzymes in glycolysis and the PPP (e.g., phosphofructokinase, PKM2, G6PD), thereby promoting the conversion of glucose to pyruvate. In aged mouse models, curcumin is associated with the expression of GDF-9, BMP-15, and glutathione, reduced ROS levels, and improves glucose metabolism. These changes correlate with increased the numbers of follicles at all stages, enhanced fertilization and blastocyst formation rates, and ameliorates reproductive hormone profiles, which may potentially delay the ovarian aging process in mouse oocytes [143]. Quercetin reduces the activity of glycogen synthase glycogen synthase kinase-3β in ovarian tissue of rats, alleviates oxidative stress, and inhibits cell apoptosis and follicular atresia [144].

#### Modulating metabolite-driven PTMs

Therapeutic targeting of glucose metabolite-driven PTMs offers a promising strategy to correct disease-associated epigenetic dysregulation and aberrant cell signaling, thereby restoring glucose metabolic homeostasis. Lactylation, a PTM linking metabolism to epigenetic reprogramming, has been shown to enhance oocyte competence. In mouse oocytes at the germinal vesicle breakdown stage, sodium lactate intervention elevated the levels of H3K14la, H4K12la, and H4K5la, promoting oocyte maturation and subsequent embryonic development. KEGG pathway enrichment analysis indicated that the differentially expressed genes were primarily involved in folate biosynthesis, OXPHOS, and glycolysis [145]. Furthermore, melatonin improved GCs function by upregulating H3K18la, increasing lactate concentration to promote glycolysis, and concurrently inhibiting ferroptosis and oxidative stress, thereby mitigating mice ovarian damage [146]. Targeting deacetylation and desuccinylation also demonstrates significant therapeutic potential. In POI, resveratrol activates SIRT1 to alleviate oxidative stress in GCs, enhance mitochondrial OXPHOS, and suppress apoptosis [147]. Its combination with melatonin further upregulates SIRT1 expression, leading to the deacetylation of its downstream target FOXO3a, increased expression of the anti-apoptotic protein Bcl-2, and reduced levels of fibrotic factors and oxidative stress. These changes collectively diminish rat ovarian fibrosis. diminishing rat ovarian fibrosis [149]. Nutritional supplement coenzyme Q10 improves oocyte mitochondrial respiratory rates and TCA cycle activity by activating SIRT1, which in turn deacetylates PGC-1α [148]. Regarding succinylation, the SIRT5 agonist MC3138 significantly reduces the succinylation level of the mitochondrial fission factor, inhibiting apoptosis in both GCs and oocytes. This suggests that elevated MFF succinylation could serve as a novel diagnostic biomarker for POI [158]. Importantly, both genetic and pharmacological activation of SIRT5 are associated with restored the recruitment and oligomerization of Dynamin-related protein 1, alongside rescued mitochondrial morphology and enhanced OXPHOS. These changes may correlate with improved oocyte quality [54, 159].

### Cell and exosome-based therapies

Cell and exosome-based therapies represent a transformative approach to ameliorating ovarian aging. These interventions have been observed to be associated with favorable changes in reproductive hormone profiles, follicle numbers, ovarian angiogenesis, key glycolytic enzyme activity, mitochondrial function, and glucose metabolic homeostasis. Recent research has increasingly focused on elucidating the network mechanisms by which these therapies intersect with metabolic regulation, indicating that their therapeutic efficacy is significantly amplified through the modulation of glucose metabolism. Stem cell therapy, particularly utilizing mesenchymal stem cells, has emerged as a forefront strategy in regenerative medicine for ovarian aging [9]. Sourced from diverse tissues such as bone marrow, umbilical cord, and placenta, mesenchymal stem cells can act as follicular precursors, activating residual primordial follicles within the ovarian cortex to restore fertility potential. In ovarian injury models, human placenta-derived mesenchymal stem cells activate the AMPK pathway and upregulate the expression of GLUT4 and insulin-like growth factor binding protein 2. These changes promote glucose metabolism, improve mitochondrial function, and increase ATP content and mitochondrial copy number. These effects are associated with improvements in glucose metabolism and ovarian function [150]. Notably, the transplantation of human umbilical cord-derived mesenchymal stem cells into patients with POI has increased the numbers of oocytes and embryos [160]. The exosomes derived from these mesenchymal stem cells are instrumental in regulating cellular senescence, apoptosis, glucose metabolic pathways, and ATP synthesis, while concurrently mitigating the inflammatory state [151]. Furthermore, therapies based on peripheral blood mononuclear cells have gained traction as a promising option for tissue and organ regeneration and angiogenesis, showing efficacy in restoring ovarian function in models of chemotherapy-induced premature ovarian failure. Similarly, platelet-rich plasma derived from peripheral blood promotes tissue regeneration. The combined administration of peripheral blood mononuclear cells and platelet-rich plasma has been shown to restore rat estrous cyclicity, increase serum levels of anti-Müllerian hormone and estradiol, elevate follicle counts, and improve implantation and live birth rates. Mechanistically, this combinatorial therapy significantly enhances follicular angiogenesis, as evidenced by increased levels of vascular endothelial growth factor, CD31, and α-SMA. Critically, it also upregulates the expression and activity of key glycolytic enzymes, including hexokinase (HK), phosphofructokinase (PFK), PKM2, and LDHA, as well as SIRT1, with the underlying mechanism being closely associated with the amelioration of glucose metabolism [152].

### Limitations and contradictions

Despite the growing enthusiasm for restoring glucose metabolic homeostasis as a strategy to delay ovarian aging, a careful examination of the existing literature reveals substantial limitations and apparent contradictions that temper the initial promise [161]. These issues span experimental models, etiological heterogeneity, translational gaps, and mechanistic ambiguities, collectively cautioning against oversimplified interpretations of metabolic “rejuvenation”. First, while restoring glucose metabolic homeostasis has shown promise in delaying ovarian aging, not all metabolic interventions yield consistent or uniformly positive outcomes across different experimental settings [162]. For example, chemically induced ovarian aging models (e.g., using D-galactose, cyclophosphamide, or lipopolysaccharide) differ fundamentally from natural aging models in their underlying pathological processes. The former typically represent acute, exogenous injury-driven follicular depletion, primarily involving apoptotic pathway activation and oxidative stress bursts. In contrast, natural ovarian aging reflects a chronic, progressive decline in ovarian reserve, involving multiple mechanisms such as mitochondrial dysfunction, chronic low-grade inflammation, and metabolic reprogramming [163, 164]. Whether metabolic interventions effective against acute injury are applicable to natural aging models remains uncertain. Second, the efficacy of these interventions appears to be highly dependent on the etiology of ovarian aging, the intervention window, and the animal model used. Furthermore, the majority of current reports are based on findings from animal studies, while evidence from human clinical research remains insufficient. The etiology of ovarian aging is complex and diverse, encompassing genetic factors, autoimmune factors, iatrogenic injury (chemotherapy or radiotherapy), and other specific causes. Under different etiological backgrounds, the metabolic profiles and signaling pathway status of the ovarian local microenvironment differ significantly, which directly affects the targets and efficacy of metabolic interventions [165]. Finally, because different animal models may respond differently to the same metabolic intervention, genetically heterogeneous mice and inbred mouse strains exhibit divergent responses to metabolic interventions. Inbred mouse strains, with their uniform genetic background, are prone to false-positive effects when assessing metabolic interventions [166]. Therefore, we emphasize that no single metabolic strategy is universally applicable, and future studies should carefully evaluate model-specific responses before drawing broad conclusions about “rejuvenation” efficacy.

Furthermore, several contradictions exist within this research field. For example, although studies have shown that treatment with the sodium-glucose cotransporter 2 inhibitor canagliflozin can prevent follicular exhaustion, it must be noted that there is little to no sodium-glucose cotransporter 2 expression in the ovary. Therefore, sodium-glucose cotransporter 2 inhibitors may exert their effects by modulating systemic metabolic homeostasis or by mediating other signaling pathways. Moreover, the effects of sodium-glucose cotransporter 2 inhibitors on oocyte quality and fecundity in mice remain unclear [167]. The efficacy of senolytics such as dasatinib and quercetin or fisetin also varies markedly across different experimental settings. In liver and kidney models, dasatinib and quercetin or fisetin have been shown to delay age-related diseases and extend lifespan in aged mice. However, long-term treatment with these agents failed to alleviate ovarian aging in a mouse model of estropause, suggesting that the ovarian microenvironment may possess unique mechanisms conferring resistance to senolytic clearance. Of note, dasatinib and quercetin treatment reduced markers of cellular senescence in obese mice, indicating that the efficacy of senolytics is highly dependent on the metabolic context [168].

### Critical evaluation of translational gaps: limitations of animal evidence for clinical prospects

Compounding these inherent limitations is the substantial translational gap between preclinical animal models and clinical application, which further challenges the practical feasibility of this approach. Although promising results have been observed in murine models for several metabolic interventions, including sodium lactate and PQQ, it must be acknowledged that they remain at the preclinical stage [60, 98, 127, 138, 141, 143, 144, 148]. To date, no such agents have a validated clinical application in assisted reproductive technology, owing to several key translational barriers. First, substantial species differences in reproductive physiology and metabolism exist between rodents and humans, including ovarian architecture, follicular dynamics, metabolic rates, and oocyte energy requirements, which may account for discordant outcomes between preclinical animal studies and human clinical applications. Second, there is a complete absence of standardized human data, as no published clinical trials or large-scale cohort studies have evaluated the safety, optimal dosing, or efficacy of sodium lactate or PQQ supplementation on oocyte quality, embryo development, or assisted reproductive technology outcomes. Third, the animal model employed in PQQ studies only mimicked chemotherapy-induced premature ovarian insufficiency (POI) and could not simulate other clinical causes; moreover, PQQ was administered prophylactically prior to chemotherapy, a regimen that does not reflect real-world clinical practice [127]. Fourth, with respect to lactylation research, dynamic changes in lactylation levels have currently only been observed during oocyte maturation and embryonic development, while the regulatory mechanisms governing lactylation in other stages of oocyte growth remain elusive, and the crosstalk between lactylation and other epigenetic modifications, such as acetylation, phosphorylation, and methylation, warrants further investigation [145]. Fifth, the relatively short intervention windows (typically 8–10 weeks) used in murine studies may not adequately model the chronic and cumulative nature of human ovarian aging, which spans decades. Finally, current clinical techniques still face difficulties in translating metabolic modulators from simple and well-controlled mouse culture systems to the complex, multi-component environment of human assisted reproductive technology laboratories. Inevitably, this transition poses unforeseen biological challenges.

Similarly, although stem cell- and exosome-based therapies have shown potential for ameliorating ovarian aging in animal studies, their clinical translation remains hindered by multiple obstacles. First, the majority of current studies are still limited to the animal level, with a lack of large-scale, well-designed human clinical trials; the depth of research on therapeutic efficacy and underlying mechanisms remains insufficient. Second, stem cell therapy raises safety and ethical concerns regarding cell sourcing, immune rejection, and tumorigenic risk, which contribute to low patient acceptance. Moreover, standardized protocols for the preparation and quality control of stem cells from different sources (e.g., bone marrow, adipose tissue, umbilical cord) have not yet been established. Third, for exosome-based cell-free therapies, data on optimal dosage, administration routes, in vivo distribution, and long-term safety remain inadequate. Collectively, the current evidence is insufficient to support the clinical application of these therapies for ovarian aging. Future efforts should focus on mechanistic elucidation, comprehensive safety evaluation, and the development of ethical frameworks.

Collectively, these limitations make it clear that we are still far from being able to recommend sodium lactate, PQQ, or similar metabolic agents in clinical practice. What we need now are well-designed, large-scale, prospective trials to figure out whether these interventions are truly safe and effective. We also need to close the gap between animal research and clinical application through rigorous trials and standardized protocols. Until solid evidence emerges, it would be wise to remain cautious about using these agents in assisted reproductive technology.

## Remaining challenges and future perspectives

Glucose metabolic homeostasis imbalance in the aging ovary is associated with multiple interrelated pathological mechanisms. These include energy disruption, dysregulation of metabolic pathways (e.g., glycolysis, OXPHOS, PPP, TCA cycle), alterations in the ovarian microenvironment (e.g., inflammation, oxidative stress, fibrosis, and vascular microcirculation dysfunction), and aberrant PTMs (e.g., lactylation and acetylation). Collectively, these factors correlate with impaired metabolic homeostasis, which may contribute to diminished ovarian reserve and reduced oocyte quality. This conceptual framework highlights the complexity of glucose metabolic dysregulation in ovarian aging and suggests that integrated approaches targeting multiple nodes may be required for effective intervention (Fig. 4). Although substantial progress has been made in understanding the mechanisms underlying glucose metabolic homeostasis, its clinical translation remains fraught with major challenges. First, from a mechanistic standpoint, restoring glucose metabolic homeostasis requires a comprehensive consideration of the spatiotemporally coordinated interplay between granulosa cells and oocytes, as well as the mediating role of the ovarian microenvironment. Metabolites in this system exhibit dual regulatory functions—acting as signaling molecules under physiological conditions while contributing to pathological cascades via PTMs, inflammation, oxidative stress, and autophagy under disease states. This functional duality underscores the limitations of broad-spectrum modulation and highlights the need for precise strategies capable of distinguishing between compensatory adaptation and maladaptive pathological reprogramming. Second, from a clinical translation perspective, pharmacological agents, stem cells, and exosome-based therapies have shown promise in preclinical models and small-scale clinical studies. However, long-term modulation of glucose metabolic homeostasis may disrupt mitochondrial quality control, impair autophagic flux, and promote multifactorial pathogenic mechanisms. These risks emphasize the necessity of rigorous long-term safety evaluations, particularly regarding administration routes, timing, and dosage—before advancing to broader clinical implementation. Third, key technological barriers persist in the accurate visualization and quantitative assessment of glucose metabolic efficiency. Conventional metabolic assays, such as immunofluorescence confocal microscopy and transmission electron microscopy—are constrained by limited resolution, phototoxicity of fluorescent dyes, and restricted access to specialized instrumentation. Encouragingly, recent advances in stable isotope tracing, single-cell metabolomics, and real-time metabolic imaging now enable targeted modulation of metabolic nodes in specific ovarian cell populations. Equally promising, such tools hold the potential to elucidate the crosstalk mechanisms that may shape therapeutic outcomes. Looking forward, future research should prioritize the integration of technological innovation with clinical translation to bridge the gap between preclinical models and practical applications. Mechanistically, glucose metabolism in the ovary should no longer be viewed solely as an energy substrate, but rather as a dynamic regulator of redox homeostasis, immune-inflammatory modulation, and epigenetic programming. However, it is currently unclear which specific signaling pathway exhibits the greatest response variability across different models of ovarian aging. The existing evidence does not permit a definitive ranking of pathway-specific heterogeneity, largely due to substantial variations in experimental conditions (e.g., animal strains, induction agents, treatment regimens, and outcome measurements) across published studies. Systematic cross-model comparisons of pathway-level heterogeneity in ovarian aging remain scarce. Therefore, future studies that directly compare multiple ovarian aging models under standardized conditions are warranted to address this knowledge gap. Elucidating the cross-regulatory signaling networks involved may not only counteract the deleterious effects of metabolic dysregulation but also preserve spatiotemporal coordination among ovarian cells and restore microenvironmental homeostasis. For clinical translation, a key priority is identifying dynamic biomarkers of real-time metabolic flux; serum pyruvate monitoring may therefore offer a future non-invasive strategy for predicting oocyte aging [8]. In parallel, the restoration of regional glucose metabolic homeostasis in the ovary should be established as a measurable criterion for therapeutic efficacy. Single metabolite concentrations are insufficient to reflect glucose metabolic homeostasis. Future efforts should focus instead on glucose uptake dynamics and the systemic status of the energy metabolic chain, aiming for multidimensional regulation through integrated pathway targeting. Additionally, leveraging the dual nature of glucose metabolism, as both an energy substrate and a signaling mediator, may offer viable clinical strategies to promote synergistic modulation within the metabolic network. On the technological front, the integration of advanced metabolic imaging platforms with artificial intelligence and big data–driven analytics holds promise for enabling precise, real-time visualization of glucose metabolic states at the single-cell level. Given the compartmentalized metabolic characteristics of oocytes and GCs, future research should actively employ single-cell metabolomics technologies to elucidate the dynamic metabolic differences between these two cell types. Large-scale clinical studies will be essential to generate high-quality evidence for updating clinical guidelines. Ultimately, the synergistic advancement of mechanistic research, emerging technologies, and translational medicine will accelerate the clinical implementation of glucose metabolic homeostasis as a therapeutic paradigm in ovarian aging.

## Data Availability

No datasets were generated or analysed during the current study.

## Abbreviations

GCs: granulosa cells
POI: premature ovarian insufficiency
DOR: diminished ovarian reserve
OXPHOS: oxidative phosphorylation
PTMs: post-translational modifications
GLUT1: glucose transporter 1
GLUT4: glucose transporter 4
TCA: tricarboxylic acid
ATP: adenosine triphosphate
PPP: pentose phosphate pathway
GDF-9: growth differentiation factor 9
BMP-15: bone morphogenetic protein 15
AGEs: advanced glycation end products
PGC-1α: peroxisome proliferator-activated receptor gamma coactivator 1-alpha
LDHA: lactate dehydrogenase A
AMPK: AMP-activated protein kinase
mTOR: mechanistic target of rapamycin
NLRP3: nucleotide-binding oligomerization domain, leucine-rich repeat and pyrin domain containing 3
HK: hexokinase
PQQ: pyrroloquinoline quinone
PDH: pyruvate dehydrogenase complex
